# The New Version of the ANDDigest Tool with Improved AI-Based Short Names Recognition

**DOI:** 10.3390/ijms232314934

**Published:** 2022-11-29

**Authors:** Timofey V. Ivanisenko, Pavel S. Demenkov, Nikolay A. Kolchanov, Vladimir A. Ivanisenko

**Affiliations:** 1 Kurchatov Genomics Center, Institute of Cytology & Genetics, Siberian Branch, Russian Academy of Sciences, Prospekt Lavrentyeva 10, Novosibirsk 630090, Russia; 2 Institute of Cytology & Genetics, Siberian Branch, Russian Academy of Sciences, Prospekt Lavrentyeva 10, Novosibirsk 630090, Russia; 3 Faculty of Natural Sciences, Novosibirsk State University, St. Pirogova 1, Novosibirsk 630090, Russia

**Keywords:** text-mining, ANDDigest, ANDSystem, named entity recognition, machine learning, PubMedBERT

## Abstract

The body of scientific literature continues to grow annually. Over 1.5 million abstracts of biomedical publications were added to the PubMed database in 2021. Therefore, developing cognitive systems that provide a specialized search for information in scientific publications based on subject area ontology and modern artificial intelligence methods is urgently needed. We previously developed a web-based information retrieval system, ANDDigest, designed to search and analyze information in the PubMed database using a customized domain ontology. This paper presents an improved ANDDigest version that uses fine-tuned PubMedBERT classifiers to enhance the quality of short name recognition for molecular-genetics entities in PubMed abstracts on eight biological object types: cell components, diseases, side effects, genes, proteins, pathways, drugs, and metabolites. This approach increased average short name recognition accuracy by 13%.

## 1. Introduction

Finding relevant information in scientific publications and patents is a significant issue when performing almost any scientific research. The number of scientific publications only in the biological sciences field has reached colossal proportions. For example, >34 million articles are stored in the PubMed database, and >1 million new biomedical articles appear annually. Modern scientific information search engines, such as those used by Google Scholar, Scopus, and PubMed [1,2,3], make it possible to find literature based on queries compiled through user-specified keywords. However, such systems do not provide practical tools for automatically extracting information from their search results, which can sometimes reach tens to hundreds of thousands of documents. In addition, they do not sufficiently consider the synonymy of the desired objects and their relationship with external databases.

Another strategy is to use programs based on automatic text analysis methods. Such systems automatically extract knowledge from documents and present it in graphical forms, such as semantic networks. Of particular interest are systems providing the full knowledge engineering cycle. This cycle includes automatic knowledge extraction from unstructured texts in natural language and external databases. It also includes integrating the obtained materials into the knowledge base as semantic networks, where nodes are the objects recognized in the texts, and the edges are the various established interaction types between them. In addition, such systems usually provide tools for the visualization and analysis of the obtained results.

STRING [4], Pathway Studio [5], MetaCore [6,7], and ANDSystem [8,9] are well-known examples of these systems. Unlike simple search engines, these programs are based on predefined and well-validated ontologies that describe the subject area. This approach automatically considers object synonymy and relationships with external databases but limits the programs’ search capacities by the size of the ontologies used. However, since their primary purpose is to establish interactions between entities and reconstruct associative networks based on the retrieved information, such tools do not attain high completeness values in finding documents or, in some cases, do not supply the user with such information.

We previously developed the ANDDigest information retrieval tool [10]. It was designed to find biomedical abstracts using complex search queries to PubMed, combining the ANDSystem ontology based on dictionaries with user-provided keywords. The ANDDigest system automatically extracts knowledge from scientific publication texts and includes tools for automated literature search and analysis.

One essential automatic text analysis step is named entity recognition (NER). A well-known problem of automatic biological entity name recognition in scientific publication texts by use of the dictionary relates to linguistic ambiguities associated with the intersection of object names with commonly used words and phrases, including abbreviations and various terms introduced directly by the authors [11]. This problem is especially relevant in biology and biomedicine for short gene and protein names [12,13,14] but is also typical in other fields [15,16]. Existing methods for overcoming the NER problem are based on three approaches: dictionaries, semantic–linguistic rules, and machine learning algorithms.

To date, machine learning algorithms are the most widespread method used. For example, the POSBioTM-NER system [17] uses named entity recognition based on support vector machines and a conditional random field (CRF). Chang et al. [18] used the vectorization methods of biomedical terms (word embedding) when training their CRF model. It allowed them to significantly increase the recognition accuracy of named entities in biomedicine compared to other approaches. Wei et al. proposed a combined machine learning method involving CRF and a bidirectional long-short-term memory network [19]. This implementation showed better results than classical methods based on rules and templates and CRF-based systems. A similar approach was used in the HUNER system [20].

A turning point in natural language processing (NLP) was the invention of the transformer neural network architecture [21]. One significant difference between transformers and previous architectures was the lack of dependence on input sequence order during training, providing ample opportunities for data parallelization and facilitating transformer models trained on vast textual data arrays reaching hundreds of gigabytes. Therefore, this architecture enabled the development of many pre-trained language models with multi-million and even multi-billion machine learning parameters, such as Megatron [22], GPT [23], and BERT [24]. Another essential feature of such models that distinguishes them from earlier machine learning algorithms such as word2vec [25] and GloVe [26] was their ability to generate context-sensitive word embeddings. The central concept is that the numerical representation for the same word (its vector) is not static but depends on its context. This point is significant since the meaning of a word can often change as a sentence or the whole text develops.

Another equally significant feature is the ability to perform additional transformer model training. This fine-tuning involves training one additional output layer while keeping the main model weights, reflecting the relationships between words, the same. This approach enables pre-trained models to be quickly adapted to solve diverse NLP tasks, including NER, relation extraction, and context-based object classification.

However, the main disadvantage of most pre-trained models is that they are trained in the general language domain, leading to insufficient accuracy when they are used to analyze texts from narrowly focused fields, such as biology and biomedicine. This problem mainly reflects additional linguistic ambiguities associated with the specifics of the biological and biomedical scientific language, which contains many highly specialized terms and abbreviations [11,27].

Another BERT model appeared in 2020, entitled BioBERT [28]. It used the classic BERT model [24] trained on the data from BookCorpus [29] and Wikipedia [30], with further pre-training on open-access biomedical texts from PubMed and PubMed central. BioBERT’s authors showed that it was more accurate for NLP tasks in biology and biomedicine than models trained on the larger textual corpora belonging to the general language domain.

Davagdorj et al. developed a BioBERT-based K-means model [31] that provided better biomedical document clustering accuracy than other models. The CPRiL web service [32] uses the BioBERT machine learning model to determine the functional relationships between small molecules and proteins in biomedical literature. This product’s harmonic mean of the precision and recall (*F*_1_) score was 84.3%, reflecting 82.9% accuracy and 85.7% recall. The STRING system’s authors used a fine-tuned BioBERT model to classify gene and protein names identified by their text-mining method in texts as correctly or wrongly recognized [4].

However, the BioBERT model’s main disadvantage was that its training used the original BERT model’s weights as the starting point. Therefore, the word embedding vocabulary was the same as the BERT model, which is specific to the general language domain and not very representative of the biomedical field.

This problem led to the development of the PubMedBERT model [33], trained from scratch using only PubMed data, with its biomedicine-specific thesaurus containing about 30% more specific terms than BioBERT. A comparative study showed that it performed best in the biomedical domain [34].

When using machine learning NER methods, one important task is establishing links between objects identified in texts and external databases containing additional information about them. A simple name comparison is often ineffective due to synonymy. A possible solution to this problem is to combine modern machine learning approaches with classical text analysis methods [4,35], such as predefined ontologies.

In this study, we developed a new version of the ANDDigest information retrieval system (ver. 01.2022) with improved short molecular-genetic object name (≤4 characters) recognition accuracy in PubMed texts. Further trained PubMedBERT models were used to filter incorrectly recognized names mapped using ANDSystem dictionaries. We used the developed models to classify object names as correctly and incorrectly recognized based on their context in abstract texts. The classification models filter eight object types: cell components, diseases, side effects, genes, proteins, pathways, drugs, and metabolites. The developed filtering methods improved recognition accuracy for these entities by 13% on average.

## 2. Results

### 2.1. Web-Based Information Retrieval System ANDDigest (Ver. 01.2022)

**Figure 1:**
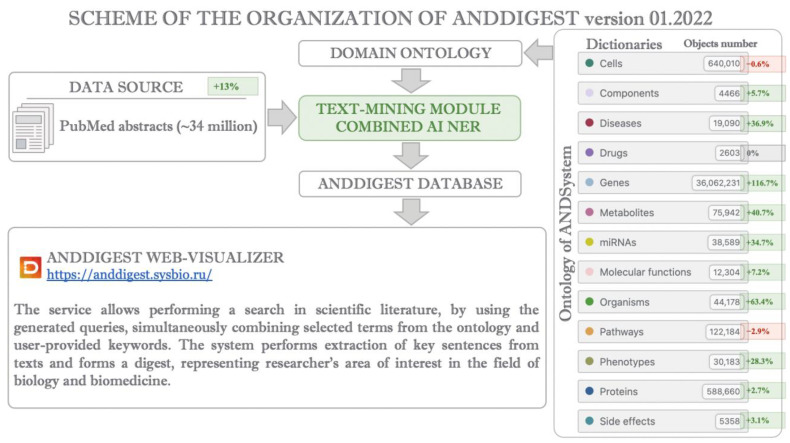
A schematic illustration of ANDDigest ver. 01.2022. Green and red colors highlight new modules and data changes compared to the previous version.

The previously developed ANDDigest software and information system [10] were designed to search and analyze information in scientific publications using a customized domain ontology. Its new version also uses an ANDSystem cognitive system ontology specific to biology and biomedicine. The general ANDDigest ver. 01.2022 module scheme is shown in Figure 1.

ANDDigest ver. 01.2022 uses the ANDSystem’s domain ontology based on dictionaries for 13 molecular-genetic object types (cells, components, diseases, drugs, genes, metabolites, micro RNAs (miRNAs), molecular functions, organisms, pathways, phenotypes, proteins, and drug side effects). Each dictionary contains the main molecular genetic object names and synonym sets.

A search query to ANDDigest can be performed by selecting specific biological object names from the corresponding dictionaries or only their types. In addition, the user can enter additional clarifying keywords. Search queries automatically consider all synonyms of the entered object. The search is performed using all objects from the corresponding dictionary when the user specifies the object type but not its name.

Search results are presented as a set of mapped texts containing the specified entities from the domain ontology and a graph of semantic relationships between their objects. In addition, the system provides flexible filtering and sorting functions for the identified documents, including filtering by the statistical significance level of the semantic relationships between pairs of objects, the impact factor of a scientific journal, and the publication date.

ANDDigest can calculate trends, indicating the scientific community’s interest in the specific object from ANDSystem’s ontology based on its number of mentions in PubMed, using the non-parametric Mann–Kendall test [36,37]. Such dynamics are calculated in two ways: (a) standard—the total number of documents mentioning the mapped object per year; (b) normalized—the ratio of the number of documents mentioning the object to the total number of published documents per year.

A new combined NER module is implemented in ANDDigest ver. 01.2022, namely combined artificial intelligence (AI) (Figure 1). This module integrates dictionary-based NER and filtering of incorrectly recognized short object names using context-based classification, which is performed by fine-tuned PubMedBERT transformer neural networks.

### 2.2. Context-Based Classification of Incorrectly Recognized Objects

Classification models were constructed by fine-tuning the pre-trained PubMedBERT transformer model [31,33] for the sequence classification task using Python’s transformers v4.16.2 library [38] with an AdamW optimizer [39] and 2 × 10^−5^ learning rate. All the texts were in lowercase, and the maximum sequence length was limited to 512 words, a standard value for BERT-based models. Each classification model was trained for 3 epochs as a binary classifier. The peculiarity of the chosen model was that it was trained from scratch exclusively on PubMed abstracts. This neural model was fine-tuned for each of the groups of objects being considered to classify short names as correctly and incorrectly recognized based on the context in which the authors mention them in their texts.

**Figure 2:**
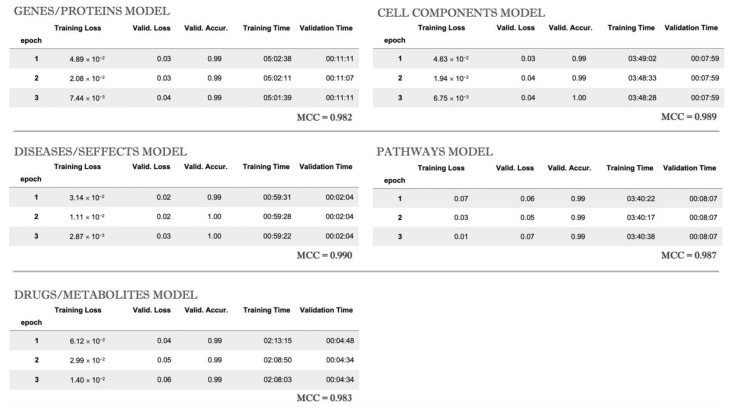
The classification model’s training results for each selected group.

After the third epoch, each classifier’s accuracy for long names was estimated by calculating Mathew’s correlation coefficient (MCC). The training results for each model are shown in Figure 2.

All negative sets were created using long object names of a single selected type for each classifier. Therefore, they could potentially cause the classifiers to perform well at distinguishing short gene and protein names from those specific types but not others. However, publicly available manually curated gold standard datasets only partly cover objects considered in the ANDSystem’s ontology [9]. Therefore, gold standards for each type were manually constructed from ANDSystem’s dictionary mapping (see Supplementary File S1) to validate the obtained classifiers on the short object names (≤4 characters) of the corresponding types. Each corpus contained a short object name mapped by ANDSystem, its position in the sentence, the corresponding abstract’s PubMed ID, year of publication, the sentence from which it was extracted, and a label indicating whether it was correctly or incorrectly identified.

The classification accuracy of short object names with the developed models was assessed using a developed gold standard that contained molecular genetic entity names from the ANDSystem ontology marked up in scientific article abstracts and manually annotated. In total, the gold standard contained >57 thousand unique sentences from >29 thousand PubMed abstracts in which at least one short object name (≤4 characters) was present. The following object types were considered: genes/proteins, metabolites/drugs, diseases/side effects, pathways, and cellular components. The gold standard was manually created in collaboration with experts while developing the new ANDDigest version (01.2022).

**Figure 3:**
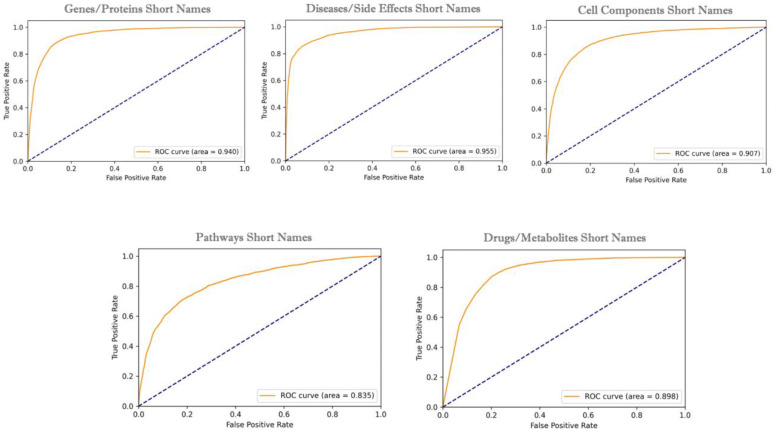
ROC curves illustrate short names’ classification accuracy of the fine-tuned models for different object types. Genes/proteins (area under the ROC curve (AUC) = 94.0%). Diseases/side effects (AUC = 95.5%). Cell components (AUC = 90.7%). Cellular pathways (AUC = 83.5%). Drugs/metabolites (AUC = 89.8%).

Each model was reassessed with the gold standard, and their accuracies for the short names recognition task were calculated using receiver operating characteristic (ROC) curves. The results are shown in Figure 3.

The optimal thresholds for positive predictions were calculated using the reconstructed ROC curves. The curve’s threshold was considered optimal when the difference between true (TPR; *y*-axis)- and false (FPR; *x*-axis)-positive rates was maximized. Detailed values for each model are provided in Table 1.

The highest accuracy (area under the ROC curve (AUC) = 95.5%) was obtained with the diseases/side effects group. This finding can be explained by the specificity of PubMed abstracts, which focus on biomedicine, and by the contextual peculiarities in how disease names are often used. The lowest accuracy was obtained with the cellular pathways group (AUC = 0.835). This finding likely reflects the very small number of short names in the cellular pathways dictionary (*n* = 61), the contexts in which pathway names are used, and the closeness of their names to common words.

The calculated ROC curves and AUC values for each model after deleting the repeating names within the same sentence are provided in Supplementary File S2. The change in AUC values compared to the full gold standard was no more than 0.001, except for the genes/proteins model, for which the decrease was 0.023.

**Figure 4:**
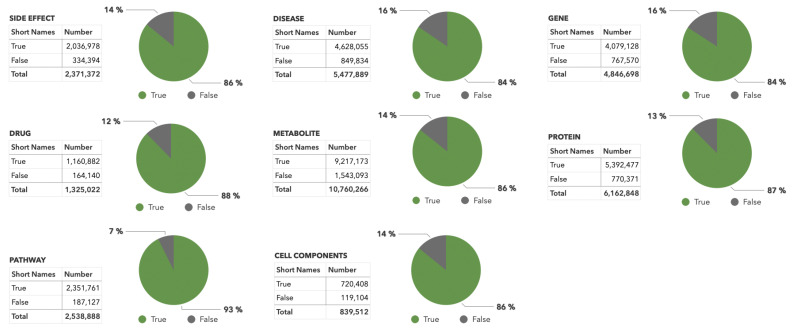
Proportions of correctly and incorrectly recognized short object words in PubMed texts after dictionary-based mapping and filtering with the developed fine-tuned neural networks. Short object names’ absolute numbers and proportions are based on their correctly or incorrectly recognized classification.

The calculated thresholds for the developed neural networks were used to analyze the PubMed abstracts previously mapped by ANDSystem. Only abstracts containing short object names of corresponding types were considered (>10 million total documents). Statistics on the obtained results are shown in Figure 4.

The results of the additional verification of the accuracy of developed models, based on the well-known gold standards, are shown in Table 2, and the ROC curves are available in Supplementary File S3. It should be noted that in general, despite the smaller number of examples for short names of cell components and pathways, the obtained values correlate with the results obtained using our own gold standard, developed with the ANDSystem ontology. The obtained results indicate the possibility of standalone application of the developed models to mapped-out document texts, including full-text articles.

## 3. Discussion

We developed a new version of ANDDigest (ver. 01.2022; Figure 1) incorporating a new combined text-mining AI NER module. The new module performs dictionary-based object mapping and filtering of short names erroneously recognized in texts.

Integration of the new module into ANDDigest ver. 01.2022 significantly increased the quality of object name recognition in texts. Due to the additional use of fine-tuned neural networks after the mapping stage, the recognition accuracy for short names (≤4 characters) increased by 13% on average. It should be noted that most recognition errors are traditionally associated with short names due to their linguistic ambiguity [11,40,41]. This problem leads to many false results when searching for relevant scientific literature based on user queries.

For example, one synonym for coproporphyrinogen oxidase is COX, which intersects with a Cox proportional-hazards model [42], widely used in biomedical literature. Therefore, even the previous version of ANDDigest identified >41,000 documents mentioning this object when searching only a smaller number of PubMed abstracts, most of which contained Cox. However, the new version recognized that most of those results were on the Cox regression model, returning only 1750 documents containing this term in the desired context after the context-based filtering.

Another example is contagious pustular dermatitis, which is a zoonotic disease caused by the *Orfviridae parapoxvirus*. One of its widely used synonyms is Orf [43]. The database of the previous version of ANDDigest contains >6400 documents, mentioning this disease. At the same time, a visual analysis showed that more than 80% of such texts were dedicated to the abbreviation of the open reading frame. Additionally, in some erroneously found documents, this term was a part of the code names of drugs, for example, Orf 12592 (5-hydroxy analog of propranolol) [44]. After applying the neural network filtering, the number of the found documents containing the Orf diseases was reduced to 562. Manual verification showed that erroneously recognized names were excluded from these documents.

The combined AI NER text-mining module performs short name recognition filtering for eight object types: proteins, genes, drugs, metabolites, diseases, side effects, cellular components, and cellular pathways. The greatest number of incorrectly identified names filtered out using this module was for genes and diseases: 16% of all recognized short names of this type (Figure 3).

The least filtered were short names of cellular pathways (biological processes). The recognition accuracy of these objects in the gold standard without the new AI NER module was about 60%; this increased to 82% with the new AI NER module. The difficulty in identifying cellular pathway names using the proposed approach can be explained by the context in which these objects occur in the text, which is very similar to objects of other types, such as diseases.

### Example Use of ANDDigest Ver. 01.2022 with Comorbid Diseases

Currently, a large proportion of the biomedical literature focuses on the problem of disease comorbidity. Comorbidity reflects the frequent joint manifestation of diseases in patients. Positive comorbidity reflects increased frequency and negative comorbidity reflects decreased frequency [45]. Our previous studies using the ANDSystem focused on molecular genetic mechanisms underlying positive disease comorbidities, such as asthma with hypertension [46,47] and pre-eclampsia associome [48]. In addition, we explored diseases with negative comorbidities, such as asthma with tuberculosis [49]. However, widely used approaches for identifying the molecular genetic mechanisms underlying comorbid diseases search for common associated genes [50,51]. In particular, we have shown that the proportion of genes simultaneously associated with two diseases is significantly higher for pairs of comorbid diseases compared to pairs of randomly selected diseases [51].

**Figure 5:**
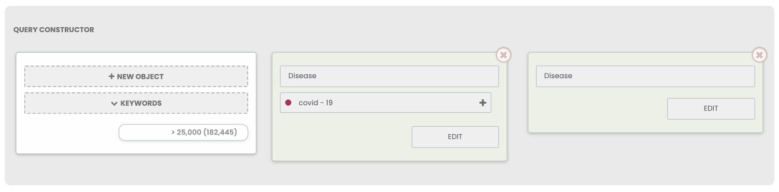
The search query used to find PubMed documents containing COVID-19 and mentioning at least one other disease with ANDDigest ver. 01.2022.

The study of the molecular genetic mechanisms for coronavirus disease 2019 (COVID-19) is extremely important in the context of the current pandemic [52]. We previously analyzed metabolomic data for the blood plasma of patients with COVID-19 using the ANDDigest and ANDSystem tools [53]. Therefore, we analyzed the comorbidity of COVID-19 with other diseases as a test case for applying the new ANDDigest ver. 01.2022 tool. The query formed to search for all documents mentioning COVID-19 and any other disease is shown in Figure 5.

ANDDigest ver. 01.2022 identified 182,445 abstracts mentioning COVID-19 and at least one of 3504 other diseases after short name filtering. Next, the resulting list of diseases was filtered based on the statistical significance of their co-occurrence with COVID-19 (false discovery rate (FDR) < 0.05) in scientific publication abstracts, identifying 84 significant diseases. The ten most common statistically significant diseases co-occurring with COVID-19 are listed in Table 3. A list of all diseases co-occurring with COVID-19, including non-significant ones, is provided in Supplementary Table S4.

Pneumonia, fever, and influenza were among the most frequently co-occurring diseases. The relationship between these pathologies with COVID-19 is widely discussed in the literature [54,55]. Interestingly, one disease significantly associated with COVID-19 in the literature was delirium (33rd on the list; see Supplementary Table S4b). Delirium is a syndrome characterized by abrupt changes in attention, awareness, and cognitive abilities. The literature discusses many factors involved in delirium’s etiology. These include neuroinflammation, cerebrovascular dysfunction, altered brain metabolism, neurotransmitter imbalance, and neural network connectivity disruption [56]. In particular, some studies report that delirium is observed in elderly patients with severe COVID-19 [57,58].

**Figure 6:**
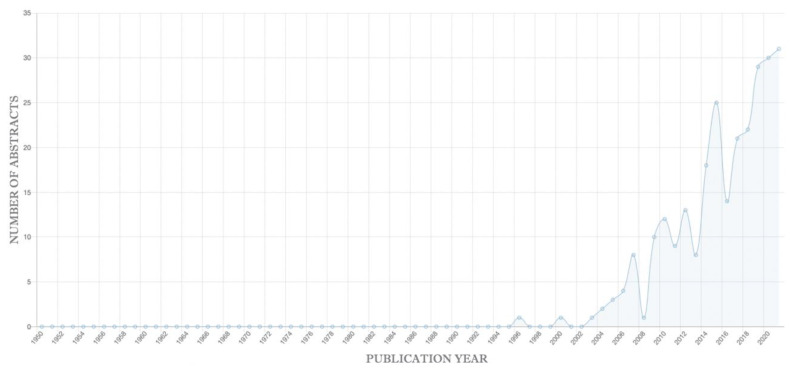
Graph showing the growth in PubMed abstracts mentioning the sigma-1 receptor by year generated with ANDDigest ver. 01.2022.

We used ANDDigest ver. 01.2022 to identify common associated genes for these two diseases using the following queries: find all publications that mention COVID-19 and at least one gene, and find all documents containing delirium and at least one gene. The first query identified 3447 genes, of which 162 significantly co-occurred with COVID-19 (FDR < 0.05). The second query identified 441 genes, of which 162 significantly co-occurred with delirium. The intersection of these two gene lists contained 230 genes common to both diseases (Supplementary File S5). They included the sigma-1 receptor (FDR (COVID-19) = 3.57 × 10^−5^; FDR (Delirium) = 6.00 × 10^−5^), which was significant for both diseases. The sigma-1 receptor has diverse functions, including regulating neuroinflammation, neurotransmitters, neurogenesis, endoplasmic reticulum stress, and mitochondrial function. The sigma-1 receptor’s significant associations with COVID-19 and delirium in the literature are consistent with its important roles in their pathologies. A graph showing the growth in publications mentioning this gene over time is shown in Figure 6.

This gene’s role in delirium has been previously discussed [59]. The role of the sigma-1 receptor as a functional host-dependency factor for the severe acute respiratory syndrome coronavirus 2 (SARS-CoV-2) virus that causes COVID-19 has also been discussed in the literature. In particular, studies have shown that the knockout or knockdown of the sigma-1 receptor causes a consistent reduction in SARS-CoV-2 replication, suggesting that the sigma-1 receptor is important in SARS-CoV-2 replication [60].

## 4. Materials and Methods

### 4.1. PubMed Abstracts Corpus

The analysis used a corpus of >34 million English PubMed abstract texts retrieved in July 2022.

### 4.2. Selection of a Maximum Length Threshold for the Analyzed Short Terms

Acronyms and abbreviations are one of the main sources of errors related to recognition of names of entities in biological literature [40,41]. At the same time, such names are present in about 15% of all PubMed abstracts, and approximately in the same proportion of clinical texts [61]. In this regard, we decided to focus on length, which is most typical for such entities when selecting a threshold value, using the corpus of abstracts, developed by Sohn et al. [62]. This corpus is the gold standard, containing 1250 randomly selected abstracts, where biomedical abbreviations were manually annotated. In total it includes 1224 names, 1121 of which correspond to unique full names. The analysis showed that 81% of all acronyms and abbreviations contained in it are terms that do not exceed 4 characters in length.

### 4.3. Dictionary-Based NER

**Figure 7:**
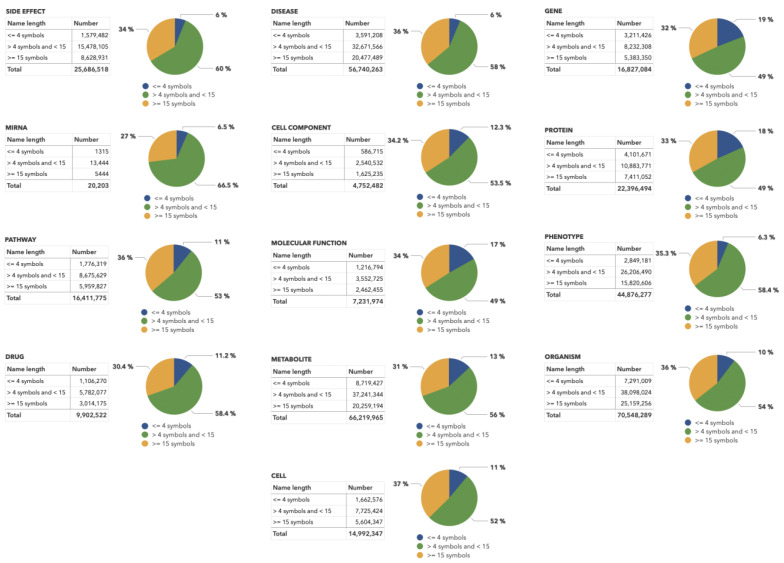
Distribution of biological object names from the ANDSystem ontology in PubMed abstracts according to their length and type. This distribution is based on the primary mapping of 34 million PubMed abstracts.

Preliminary dictionary-based mapping of the molecular genetic object names in texts is performed using the text-mining algorithms implemented in ANDSystem [11]. Then, all the recognized entities matching the corresponding dictionary are divided into three groups: terms with a length of ≤4 characters (short names), terms with a length of >4 but <15 characters, and terms with a length of ≥15 characters (long names). The distributions of object names by group, length, and type are shown in Figure 7.

The justification for filtering short names is that the most significant error associated with semantic concept ambiguity is their more frequent intersection with common words and various abbreviations [13,14,15,16]. For example, one synonym for the cyclin-dependent kinase 4 inhibitor B gene is p15. This word often occurs in texts as a page number. Another example is flu, traditionally used as a synonym for influenza. However, the UniProt database contains information on an *Escherichia coli* gene (UID: P39180) with the same name. Similarly, the *tic* term often corresponds to impaired nervous system functioning in biomedicine. However, this term was introduced as an abbreviation for tumor-initiating cells in a study on epithelial–mesenchymal transition [63].

Figure 7 shows that most references to short names in >10 million scientific publication abstracts belong to metabolites. This finding reflects the fact that this dictionary contains numerous chemical element names whose length does not exceed two letters, such as Ca (calcium), Pb (lead), and Mg (magnesium). Moreover, most of these terms also intersect with different abbreviations. For example, CA is also used as a short name for California, mg as milligrams, while in medicine, Pb can be short for peripheral blood. Another example is the name gold, which is often used in the context of the gold standard.

These examples highlight errors that might appear when using only dictionary-based mapping methods. One solution to this problem is the subsequent filtering of such dictionary-mapped entities according to their context.

### 4.4. Training Sets

Five object groups were considered: (1) proteins and genes, (2) diseases and side effects, (3) drugs and metabolites, (4) cellular components, and (5) cellular pathways. The protein and gene vocabularies were combined into a common vocabulary, as were those for diseases and side effects, and for drugs and metabolites. Our analysis did not consider organisms, phenotypic traits, miRNAs, molecular functions, and cells.

**Figure 8:**
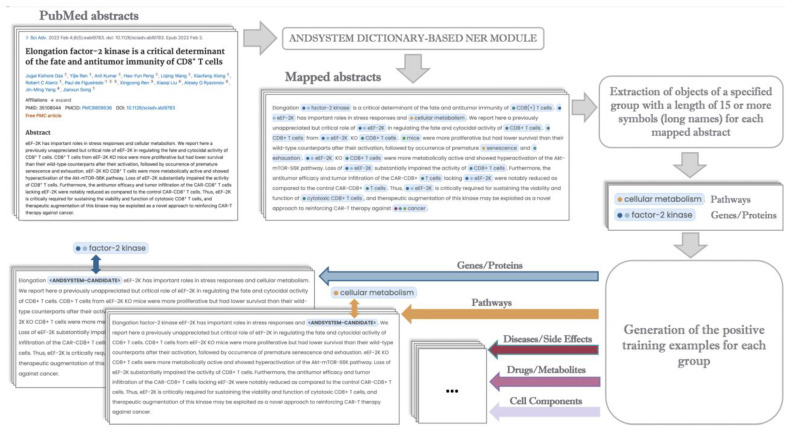
The algorithm scheme for generating training examples for fine-tuning the ANDDigest ver. 01.2022 classification models.

The automated formation of training samples for each classification model was based on the following algorithm: mapped PubMed texts containing long object names of the corresponding type from the group being considered were selected as positive examples. The mapping was performed using the ANDSystem’s ontology and text-mining approach. Terms of ≥15 characters were considered as long. The number of examples mentioning such names for each selected group exceeded 1 million (Figure 7), making it possible to use them as training sets. Often several objects can be mentioned in the text, and a given name can appear multiple times in the text. Therefore, to provide the neural network with the ability to consider the context of a particular object in the specific part of the sentence, the classified term was separately replaced by a special tag: <ANDSYSTEM-CANDIDATE>. A schematic illustration of the algorithm is shown in Figure 8.

Positive examples for objects of another group were used as negative examples. Therefore, for the drugs/metabolites and diseases/side effects groups, data from the genes/proteins group were used as negative examples, while for the genes/proteins, cellular components, and cellular pathways groups, data from the diseases/side effects group were used as negative examples. Each model’s learning set comprised 512,000 training and 50,000 validation examples, with positive and negative examples in a 1:1 ratio. All classification models were trained on the context of objects with ≥15 characters.

### 4.5. Gold Standards

To assess the accuracy of the classification models, in relation to names of the groups being considered from the ANDSystem ontology, where the length did not exceed the selected threshold value, a gold standard was prepared containing positive and negative examples of dictionary-based recognition.

The process of preparing the gold standard included the following steps: at first, the PubMed texts were automatically downloaded in blocks from their official ftp server. Then, using the previously implemented ANDDigest and ANDSystem pipeline, each block of data was automatically pre-processed, which included converting texts into a unified format, their normalization, removing duplicating texts, and dictionary-based mapping of objects. Next, for each model, we randomly selected a block of pre-processed data, where, according to the groups of objects selected for classification, all abstracts containing short names, whose length did not exceed 4 characters, were allocated.

The obtained mapped texts for each group of objects were manually analyzed by a single specialist. At the same time, since some of the sentences contained repeated short names, an additional variant of corpuses was prepared from which such repetitions were excluded.

For an additional assessment of the accuracy of each classification model, we used the existing gold standards containing manually mapped objects, with types that intersected with the ANDSystem ontology (Table 4).

Using the selected corpuses, for each model, 2 groups of positive and negative examples were formed in a 1:1 ratio. In the first case, all lengths of annotated object names were used as positive examples. In the second, only short names were considered. For negative examples, the names of entities from gold standards corresponding to other object types were taken, excluding positive ones for each corresponding model. The process of generating examples was carried out with the same algorithm used for the preparation of training samples (Figure 8).

As positive examples for the disease and side effects model, the mapped names corresponding to the DiseaseOrPhenotypicFeature type from the BioRED corpus were used: 5545 examples were generated for names of any length and 1127 for short names only. These examples were expanded with texts, containing the disease entities’ names from the NCBI Disease gold standard: 4953 and 1040 examples, respectively. To generate negative examples, a similar number of all other types of objects from the BioRED dataset was used, and the objects were selected consecutively, according to their mention in the text.

Names mapped as chemical entities from the BioRED corpus, were used to generate positive examples for the drug/metabolite classifier. The corpus enabled the creation of 4429 examples for any name length and 1080 only for short ones. These data were expanded with information from the NLM-Chem corpus. Due to the fact that NLM-Chem is a gold standard built using the full texts of articles, its separate blocks, enclosed inside <text></text> tags with a mapped chemical compound, were used as a context. All blocks that did not exceed 250 characters in length were excluded. Based on NLM-Chem, 11,561 more positive examples were generated for all lengths and 3731 for short names only. Negative examples were formed using the BioRED corpus, in the same way as the previous model.

For the gene/protein classification model, all tagged texts from the BioRED gold standard, containing objects mapped with a GeneOrGeneProduct type, were used. This enabled the generation of 6697 positive examples for all lengths and 2859 for short names. The obtained data were expanded with information from the CRAFT corpus, where each line with a length of at least 250 characters and with at least one tagged gene or protein, was considered as a context. Using the CRAFT corpus, 5358 more positive examples were added, based on names of any length, and 2084 based on only short ones. Negative examples for objects of any length were formed using the BioRED corpus, while for short names, it enabled the generation of only 2946 examples and was also expanded using the other datasets. The expansion was achieved by adding more examples containing short names corresponding to Diseases (NCBI Disease), Cellular Pathways (CRAFT), and metabolites (NLM-Chem). In each case, 680 first records were used.

For the classification models for cellular pathways and cellular components, positive examples were formed using the corresponding sections from the CRAFT corpus, while the negative examples were generated with the BioRED dataset. This enabled the creation of 774 positive examples for the short terms, and 12,885 for terms of any length. For cellular components, these values were 811 and 4441, respectively.

## 5. Conclusions

We have shown that the developed AI NER text-mining module integrated into ANDDigest ver. 01.2022 has high efficiency in recognizing short-named entities. A feature of the new ANDDigest version is the use of neural networks that perform binary classification of short names for biological objects in the ANDSystem ontology based only on the context in which they are mentioned. This approach makes it possible to overcome linguistic ambiguities inherent to general dictionary-based text mapping methods and the previous ANDDigest version in particular. In addition, we showed the effectiveness of our automated generation of high-quality training samples based on the context of long names for various object types. Moreover, preliminary dictionary mapping provides the user with all the necessary information about the recognized entities, such as their synonyms and links to external databases.

## Figures and Tables

**Table 1:** TPR, FPR, and optimal thresholds for each classification model.

Group	TPR	FPR	Optimal Threshold (Positive)
Cellular components	0.85	0.17	0.9999737739562988
Diseases/side effects	0.85	0.08	0.9999943971633911
Genes/proteins	0.89	0.13	0.9999139308929443
Cellular pathways	0.80	0.21	0.9998261332511902
Drugs/metabolites	0.89	0.20	0.9999928474426270

**Table 2:** AUC values for each model, calculated using the existing gold standards for objects of any length, and for short objects only.

Classification Model	AUC All Names	AUC Short Names
Cellular components	0.919	0.906
Diseases/side effects	0.934	0.943
Genes/proteins	0.924	0.897
Cellular pathways	0.864	0.731
Drugs/metabolites	0.944	0.928

**Table 3:** The top 10 diseases that significantly co-occurred with COVID-19 in the query results.

Rank	Disease	Document Number	Co-Occurrence Score (*p*-Value)	FDR *p*-Value (<0.05)
1	Severe COVID-19	4584	2.08593 × 10^−8^	3.496523 × 10^−6^
2	Pneumonia	3944	7.00031 × 10^−8^	4.968849 × 10^−6^
3	Fever	3396	2.77321 × 10^−8^	3.506271 × 10^−6^
4	Acute respiratory distress syndrome	2600	3.20772 × 10^−8^	3.593681 × 10^−6^
5	Severe acute respiratory syndrome	2573	1.65303 × 10^−8^	3.496523 × 10^−6^
6	Infectious diseases	2524	3.42188 × 10^−8^	3.627905 × 10^−6^
7	Influenza	2431	3.36938 × 10^−9^	2.689255 × 10^−6^
8	Viral infection	2336	3.68056 × 10^−8^	3.627905 × 10^−6^
9	Breathlessness	1935	4.75682 × 10^−8^	4.236009 × 10^−6^
10	Fatigue	1738	1.01457 × 10^−8^	3.203274 × 10^−6^

**Table 4:** List of the well-known gold standards, used for the additional evaluation of the accuracy of the fine-tuned classification models.

Gold Standard	Description	Types of Objects Considered	Reference
BioRED	Rich biomedical relation extraction dataset (BioRED), containing several types of molecular-genetics entities and their relationships, expertly labeled in a corpus of 600 PubMed abstracts.	Disease/Side effects, Drugs/Metabolites, Genes/Proteins	[64]
NCBI Disease corpus	The corpus is made of 793 fully annotated PubMed abstracts, containing 6892 disease mentions, mapped to 790 unique concepts.	Disease/Side effects	[15]
NLM-Chem	The NLM-Chem corpus contains 150 full-text articles with over 5000 unique chemical names, annotated by ten expert NLM indexers.	Drugs/Metabolites	[65]
CRAFT	The Colorado richly annotated full-text corpus contains 97 full-text biomedical articles, annotated by using the nine biomedical ontologies and terminologies.	Cell pathways, Cell components, Genes/Proteins	[66]

## Data Availability

The new ANDDigest version (01.2022) has a web interface and is freely available at https://anddigest.sysbio.ru/ (accessed on 11 November 2022). The fine-tuned classification models and datasets are available upon request at the following link: https://huggingface.co/Timofey (accessed on 11 November 2022). Codes and examples for standalone training and application of the fine-tuned models, gold standards and output results for each corresponding model, are available at GitHub: https://github.com/ANDDigest/ANDDigest_classification_models (accessed on 11 November 2022).

## Supplementary Materials

The following supporting information can be downloaded at: https://www.mdpi.com/article/10.3390/ijms232314934/s1.
