# Supplementary material for: The New Version of the ANDDigest Tool with Improved AI-Based Short Names Recognition
Source: Int J Mol Sci. 2022 Nov 29;23(23):14934. doi: 10.3390/ijms232314934 (PMC9738852; doi:10.3390/ijms232314934)

ROC curves illustrating short names' classification accuracy of the fine-tuned models for the selected groups of object types, after removal of repeating names within the same sentence, obtained using the developed Gold Standard from the Supplementary File S1

Genes/Proteins

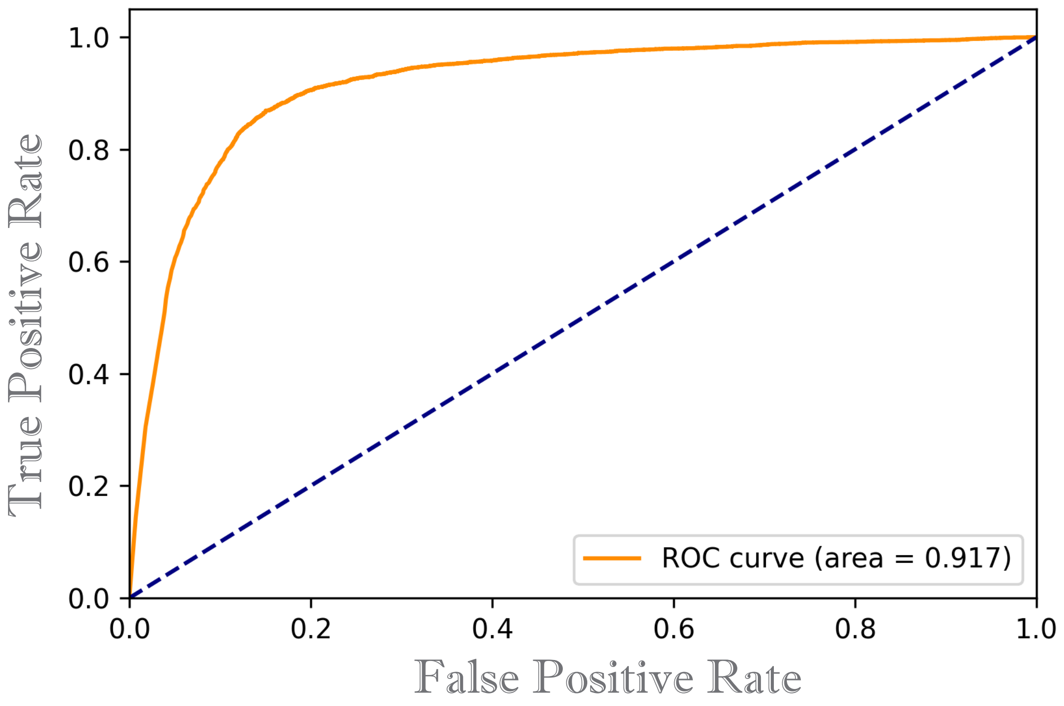

Drugs/Metabolites

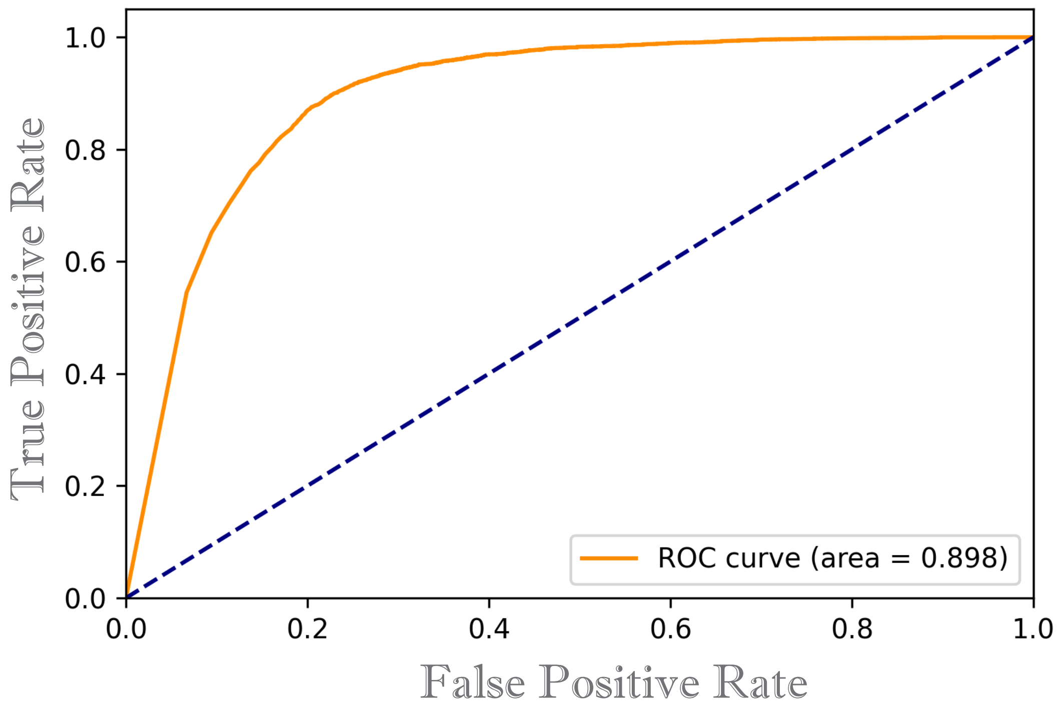

Diseases/Side Effects

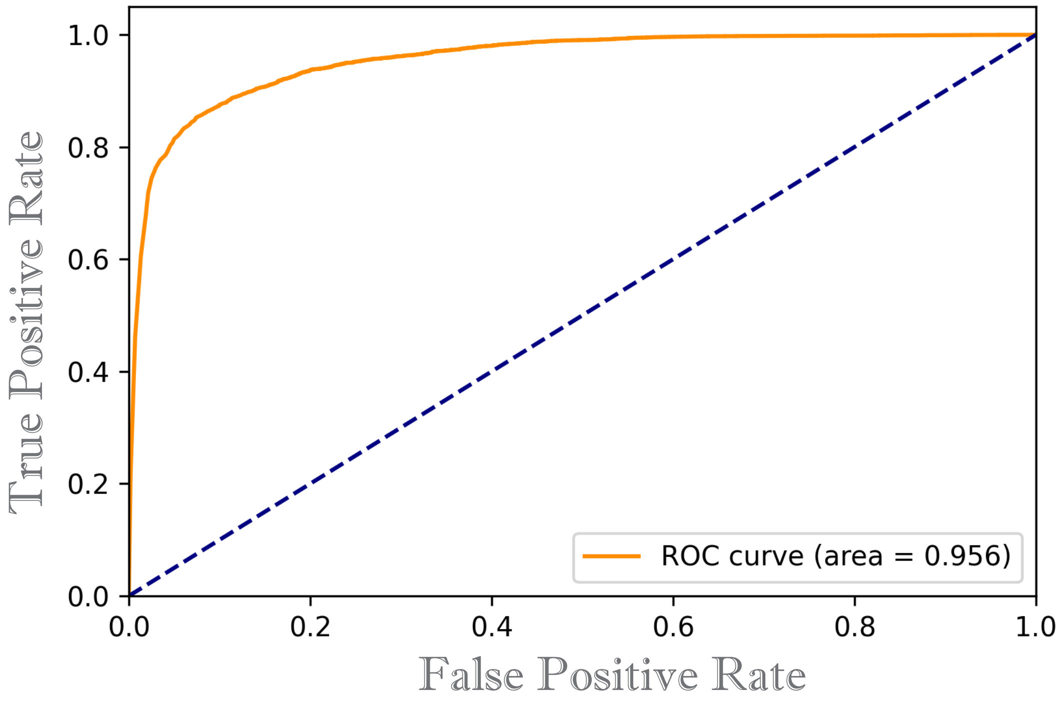

Pathways

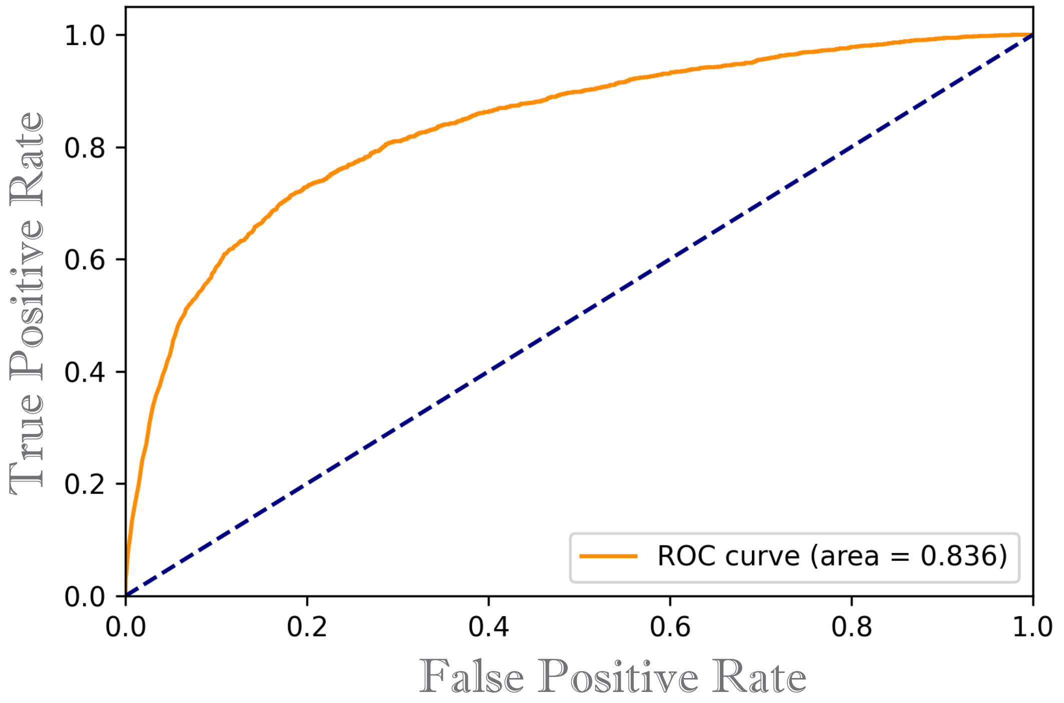

Cell Components

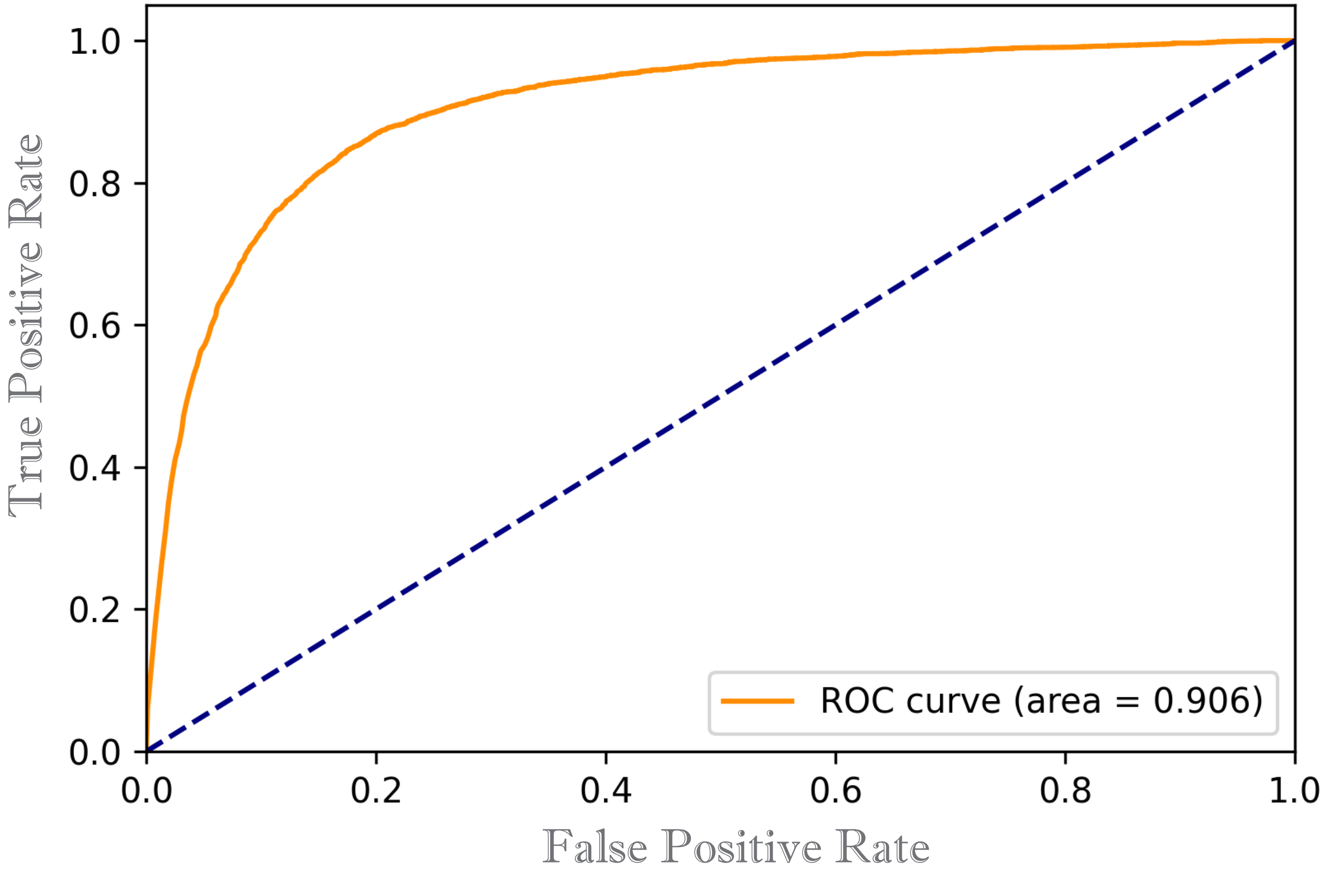

Supplement: Supplementary file 1 [file ijms-23-14934-s001.zip › Supplementary_File_S2.pdf]
