# Supplementary material for: The New Version of the ANDDigest Tool with Improved AI-Based Short Names Recognition
Source: Int J Mol Sci. 2022 Nov 29;23(23):14934. doi: 10.3390/ijms232314934 (PMC9738852; doi:10.3390/ijms232314934)

ROC curves illustrating classification accuracy of the fine-tuned models for the selected groups of object types, for the short names only and the names of entities of any length, obtained using the existing Gold Standards, presented in Table 1

Genes/Proteins

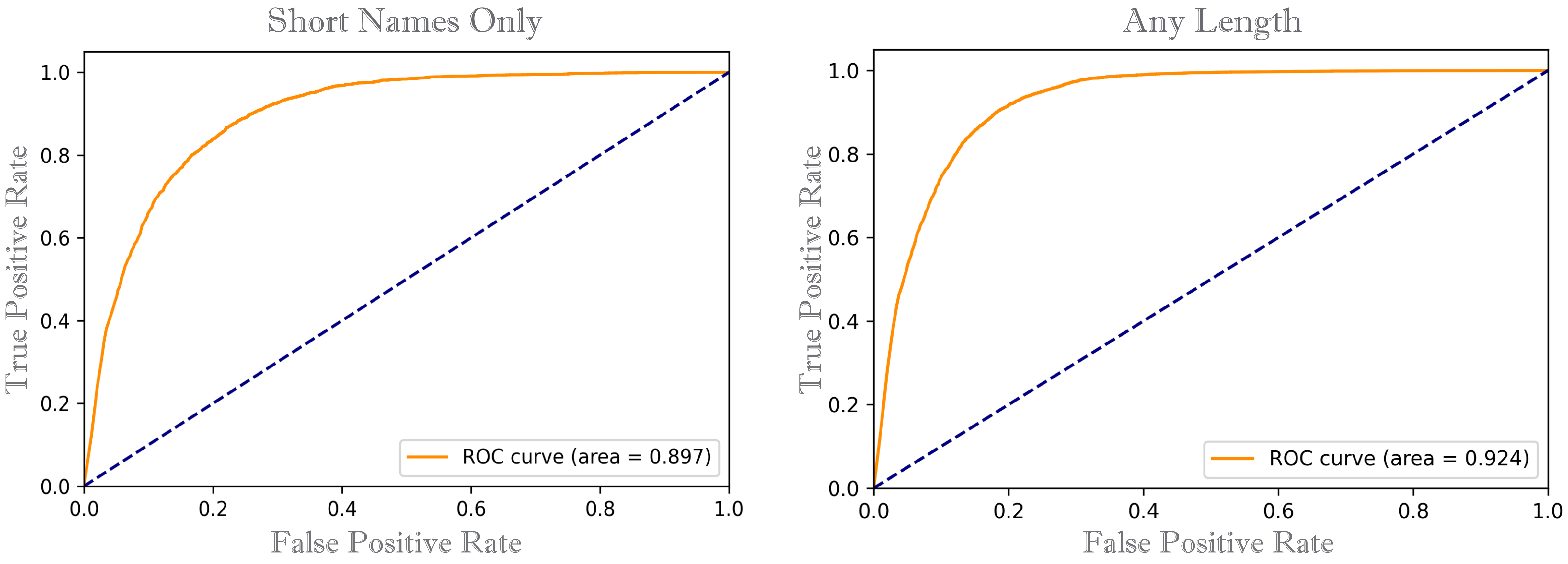

Drugs/Metabolites

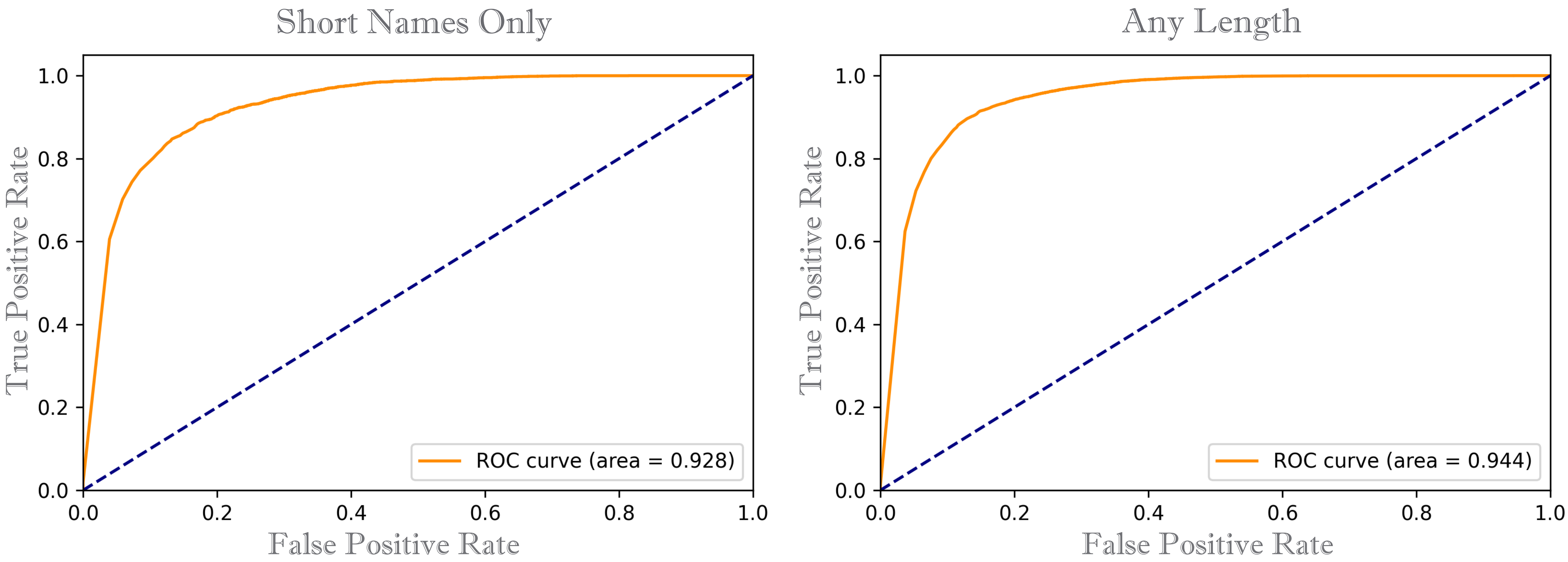

Pathways

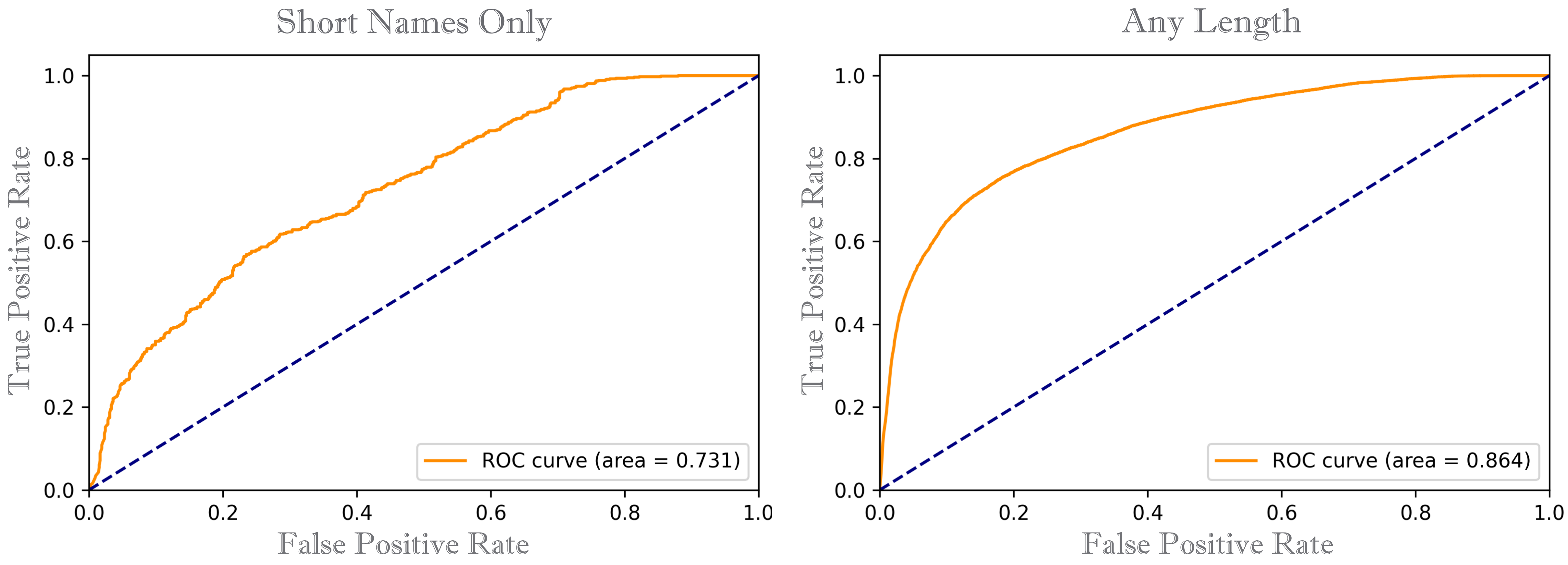
Short Names Only
Any Length
True Positive Rate
False Positive Rate
ROC curve (area = 0.731)
ROC curve (area = 0.864)
0.0
0.2
0.4
0.6
0.8
1.0

Cell Components

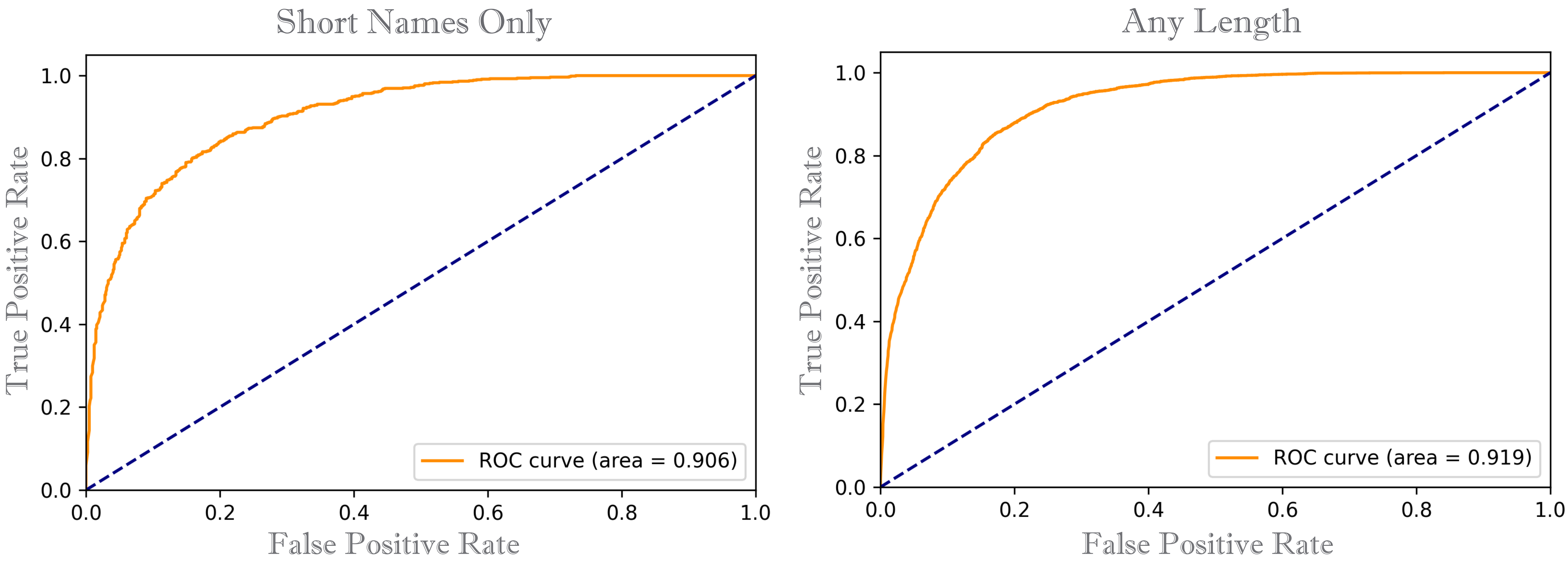

Diseases/Side Effects

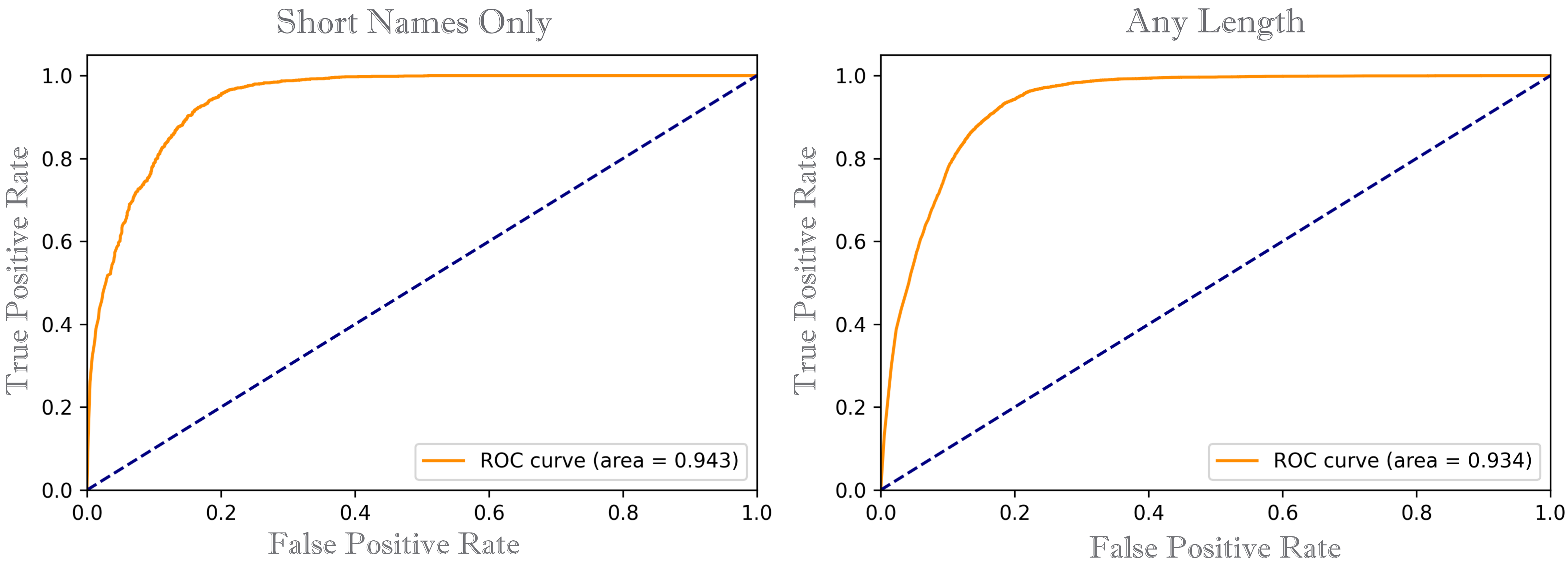

Supplement: Supplementary file 1 [file ijms-23-14934-s001.zip › Supplementary_File_S3.pdf]
